# Application of New Sources of Bioactive Substances (*Perilla frutescens* L. and *Tagetes erecta* L.) in the Chosen Cookies Production

**DOI:** 10.3390/ijerph191811504

**Published:** 2022-09-13

**Authors:** Małgorzata Moczkowska-Wyrwisz, Dominika Jastrzębska, Jarosław Wyrwisz

**Affiliations:** 1 Institute of Human Nutrition Sciences, Department of Food Gastronomy and Food Hygiene, Warsaw University of Life Sciences, 159c Nowoursynowska St., 02-776 Warsaw, Poland; 2 Institute of Human Nutrition Sciences, Warsaw University of Life Sciences, 159c Nowoursynowska St., 02-776 Warsaw, Poland; 3 Institute of Human Nutrition Sciences, Department of Technique and Food Product Development, Warsaw University of Life Sciences, 159c Nowoursynowska St., 02-776 Warsaw, Poland

**Keywords:** muffins, plant extracts, antioxidant capacity, color, texture

## Abstract

Today, one of the most important challenges of ensuring the society’s health is the prevention of civilization diseases. Most of them are associated with an imbalance between antioxidants and free radicals in the human body. Due to the need to increase the intake of antioxidants, opportunities are being studied to increase their consumption. Therefore, the aim of this study was to evaluate the influence of plant extracts of marigold (*Tagetes erecta* spp.) (MEx) and perilla (*Perilla frutescens* L.) (PEx) on selected qualitative properties of muffins. We studied the effects of the extracts in amounts of 1% (1), 3% (3), and 5% (5) on selected muffin characteristics, i.e., crust and crumb color, cooking yield, specific volume, and water activity, and changes in textural properties as well as sensory acceptance analysis. The level of crust lightness (L*) of muffins was lower than that of the control sample only for the samples with the PEx. For the crumb, the muffins with MEx and PEx were darker than the control sample. Fortification of muffins resulted in a concentration-dependent increase in antioxidant capacity. The PEx_3 and PEx_5 samples were rated highest in the sensory acceptance evaluation. The other quality attributes were similar to the control sample, indicating the validity of introducing extracts in the muffin recipe.

## 1. Introduction

Nowadays, food consumption trends focus on convenience foods that are personalized, while performing certain functions. To achieve all the requirements, technologists often use synthetic substances to meet these expectations. However, consumers are increasingly concerned about the health effects of food and ingredients due to food additive content. Therefore, it is now an observed trend resulting in several changes in the food industry. As a result, the clean label trend is becoming increasingly popular. One such solution is the orientation toward the use of bioactive plant extracts, which can be an alternative to synthetic agents added to food, for example as antioxidants to prevent the oxidation of lipids and other compounds susceptible to oxidative processes. The most commonly used raw materials are those that, in addition to their nutritional functions, have a beneficial effect on the formation and/or improvement of the quality of food products. These may be added to ensure texture stabilization and enhance sensory properties, such as marine algae [1,2,3], or provide an alternative to sucrose and synthetic sweeteners (xylitol or stevia) [4,5,6,7]. In addition, green tea extracts are often used [8,9], as well as ginger [10], thyme [11], and other spices, which are found in the kitchen, as an ingredient in many dishes and hot beverages due to their aroma and flavor and have possible antioxidant properties [12]. The addition of such extracts can favorably affect the shelf life of food products.

For thousands of years, humans have used plants as a source of food and nutrients. In addition to their primary function, many of them show beneficial effects on human health [13,14,15,16] and also show application potential in the food industry [6,8,10]. Today, plant extracts are becoming increasingly important food additives due to their content of bioactive compounds such as phenols, carotenoids, anthocyanins, and tocopherols [17]. In addition to the above-mentioned groups of raw materials, plants such as herbs, flowers, trees, legumes, and shrubs are also rich in health-promoting compounds, especially those with antioxidant activity [18]. The use of plants as natural sources of nutrients with health-promoting properties has long been cultivated in Far Eastern countries, where many plants are used in traditional dishes, such as the perilla (*Perilla frutescens* L.) or edible flowers of marigold (*Tagetes* spp.).

Marigold extracts present a rich set of chemical compounds such as rutin, isoquercitrin, quercetin-3-O-rutinosylrhamnoside, isorhamnetin-3-O- rutinosylrhamnoside, isorhamnetin-3-O-glucosylgluco-side and isorhamnetin-3-O-glucoside, caffeic and chlorogenic acids, p-coumaric acid, and many others with a variety of properties. These substances belong to the classes of secondary plant metabolites, among which are: flavonoids, sterols, carotenoids, tannins, saponins, triterpene alcohols, polysaccharides, bitterness, mucilage and resins, and essential oils [19,20]. Moreover, marigold flower extract shows antibacterial activity (especially against Gram + bacteria) [15]. It is worth emphasizing that marigold flower extracts and essential oils do not exhibit antimicrobial activity against the human microbiome, considered crucial for maintaining the body’s immunity, so its use in formulations and as a food additive can support the treatment of intestinal diseases [21]. In particular, essential oils isolated from these plants are valued ingredients and are used as functional additives in food. Marigold plant oil extracts have a wild, strong, and sweet and slightly citrus aroma [22]. On the other hand, the perilla plant is primarily used as a raw material in the production of medicines, and in the treatment of asthma, cold, cough, among others. In addition, it exhibits anti-allergic effect, anti-inflammatory, and antioxidant properties [14,16,23,24]. The perilla plant is characterized by a minty fragrance, its appearance resembles that of basil, which is why it is why it is sometimes called Chinese basil [25,26]. Due to its medicinal properties and dietary and sensory qualities, it is widely spread around the world, especially in Southeast Asian countries including Thailand, China, Korea, Japan, and Vietnam [27]. Ingredients isolated from this plant are used as a component of food preservatives, as well as a natural food colorant [26,28]. Therefore, the purpose of this study was to evaluate the effect of bioactive plant extracts from marigold (*Tagetes erecta* spp.) (MEx) and perilla *(Perilla frutescens* L.) (PEx) on selected quality characteristics of pastry products, using muffins as an example.

## 2. Materials and Methods

### 2.1. Materials

For the batter formulation, a local market supplied the dry and wet ingredients: wheat flour type 450 (10% protein, 0.51% ash, and 28.6% gluten with a 14.04% moisture content) (Lubella Food Sp. z o.o. Sp.k., Lublin, Poland), sugar (Südzucker Polska S.A., Wrocław, Poland), vanilla sugar (Master Cook Polska Sp. z o.o. Sp.k., Pruszków, Poland), baking powder (Dr. Oetker Polska Sp. z o.o., Gdańsk, Poland), salt (Ciech S.A., Nowa Sarzyna, Poland), natural yogurt (District Dairy Cooperative in Krasnystaw, Poland), rapeseed oil (Grobsol Sp. z o.o., Pruszków, Poland), eggs (Farmio S.A., Warszawa, Poland), and powdered extracts of marigold flowers (*Tagetes erecta* L.) (MEx) and perilla leaves (*Perilla frutescens*) (PEx) (PK Components Sp. z o.o. Sp.k., Warszawa, Poland). Extracts of marigold flowers and perilla leaves were produced by spray drying. The marigold extract was standardized for Lutein (Lutein 5%) while perilla extract for flavones (Flavones 2.5%).

### 2.2. Batter Preparation and Muffin Baking

Muffins were made in 7 different formulations: the control sample (without extract), 3 samples with marigold extract (MEx), and 3 samples with perilla extract (PEx). Both MEx and PEx extract were added in amounts of 1%, 3%, and 5%, replacing flour in the recipe in relation to the mass of all raw materials. To prepare the muffin batter, all dry and wet ingredients were weighed using a balance (RADWAG PS 4500.R2.M, Radom, Poland) according to the recipe (Table 1). Eggs were beaten into a bowl and mixed for 1 min, then sugar was added, and mixed until a smooth, fluffy batter without fine sugar crystals was obtained, using a mixer (Kitchen Aid model 5KPM, Springfield, Geneseo, IL, USA). Next, rapeseed oil was added and blended for 2 min, then natural yogurt was added and blended like this for about a minute. Next, dry ingredients (flour and baking powder) including extracts (depending on the recipe used—Table 1) were sifted twice, and salt was added and combined with the wet ingredients mixture. The whole mixture was still mixed for about 2 min until all the ingredients were combined. The prepared batter thus was placed into molds (50 ± 3 g) and baked for 25 min at 180 °C (Küppersbusch model CPE 110, Gelsenkirchen, Germany). Muffins were cooled at ambient temperature for approx. 1 h and then packed and stored in a polyethylene bag for 24 h. After this time, an analysis was conducted.

### 2.3. Oil-Holding Capacity (OHC) of Extracts

The OHC was conducted in triplicate according to the method described by Moczkowska et al. [29] with slight modifications. The 10% dispersions of MEx and PEx were prepared with rapeseed oil in centrifuge tubes. The 0.50 g of dried sample was mixed with 5 mL of rapeseed oil in a 12 mL preweighted centrifuged tube. The sample was allowed to equilibrate in refrigerated conditions (2 ± 1 °C) for 1 h and then was centrifuged at 3000× *g* for 20 min. The supernatant was discarded, and the wet pellet was weighted. The *OHC* was calculated using the equation below (1):

(1) $$OHC\left[\frac{g}{g}\right]=\frac{\mathrm{pellet}\;\mathrm{weight}\;\mathrm{dry}\;\mathrm{weight}-\mathrm{dry}\;\mathrm{weight}}{\mathrm{dry}\;\mathrm{weight}}$$

### 2.4. Color Measurement of Plant Extract

According to Moczkowska et al. [29], the color of MEx and PEx was carried out in a CIE L*a*b* system using a Minolta CR-400 chromatometer with granular attachment CR-A50 (Konica Minolta, Inc., Tokyo, Japan). The diameter of the measuring head was 8 mm. The device was calibrated on a white standard plate (L* = 98.45, a* = −0.10, b* = −0.13). The illuminant D65 (color temperature: 6500 K) and a standard observer (2°) were used. The determined parameters were L* [L = 0 (black) and L = 100 (white)], a* (−a = greenness and +a = redness), b* (−b = blueness and +b = yellowness). Ten measurements were conducted for each extract.

### 2.5. Cooking Yield

The cooking yield was determined according to Karp et al. [4]. For this purpose, 10 muffins from each group were weighed before baking, after baking, and after cooling. The cooking yield was calculated according to Formula (2):

(2) $$CY=\frac{W_{a}}{W_{b}}\cdot 100\%\;$$

where

*CY*: cooking yield (%)
*W_b_*: weight of sample before baking (g)
*W_a_*: weight of sample after baking and cooling (g)

### 2.6. Specific Volume

The volume of muffins was determined by the rapeseed displacement method American Association of Cereal Chemists (American Association of Cereal Chemists (AACCI method 10-05.01) [30]) and then divided by the mass of each muffin to achieve the result (cm^3^/g).

### 2.7. Water Activity

The water activity was measured by the dew point methodology using Water Activity Meter 4 TE (Aqualab, Pullman, WA, USA) according to the method described in previous studies (Wyrwisz et al. [31] and Karp et al. [4]). The measurement was performed in triplicate (*n* = 3).

### 2.8. Color

Color of muffin’s crust and crumb was performed in a CIE L*a*b* system using a Minolta CR-400 chromatometer (Konica Minolta Inc., Osaka, Japan) according to the method described by Wyrwisz et al. [31]. The diameter of the measuring head was 8 mm. The device was calibrated on a white standard plate (L* = 98.45, a* = −0.10, b* = −0.13). The illuminant D65 (color temperature: 6500 K) and a standard observer (2°) were used. The determined parameters were L* [L = 0 (black) and L = 100 (white)], a* (−a = greenness and +a = redness), b* (−b = blueness and +b = yellowness). Ten measurements (*n* = 10) were conducted in different spots of muffins. 

The overall color difference Δ*E* between the samples with extracts (x) and the control sample—sample without extract addition (0) was calculated from Equation (3):

(3) $$∆E=\sqrt{{\left(L_{x}^{*}-L_{0}^{*}\right)}^{2}+{\left(a_{x}^{*}-a_{0}^{*}\right)}^{2}+{\left(b_{x}^{*}-b_{0}^{*}\right)}^{2}}$$

### 2.9. Texture

The texture of muffins was evaluated using an Instron 5965 universal testing machine (Instron, Canton, MA, USA) with Bluehill 2 software installed. The double compression test (Texture Profile Analysis—TPA), according to the method described in previous studies by Wyrwisz et al. [31] and Karp et al. [4], was performed. The following texture components were analyzed: firmness, springiness, and cohesiveness. Samples were cut into 2 cm × 2 cm × 2 cm cubes, and double compression tests were performed in eight repetitions (*n* = 8) for each sample. A 50 mm cylindrical probe, 50% compression of the sample, and 5 s relaxation time were used. A 50 N load cell was used, and the crosshead speed was set at 120 mm/min.

### 2.10. Total Phenol Content (TPC)

Prior to the TPC and DPPH* determination, muffin extracts were prepared according to Gangopadhyay et al. [32]. Muffin (1 g) was mixed with 9 mL of 80% methanol and homogenized for 2 min at 12,000 rpm using a T18 Ultra Turrax basic high-speed homogenizer (IKA, Staufen, Germany). Samples were mixed on a rotator for 30 min with shaking (MyLab SLRM-3, NanoEnTek Inc., Seoul, Korea) at room temperature for 10 min. After that, samples were centrifuged for 10 min at 8000 g (Hettich Universal 320R, Tuttlingen, Germany). The supernatant liquid was carefully poured off from the solid pellet and analyzed for TPC and DPPH* analysis. 

Total phenolic content in the samples was determined using the Folin–Ciocalteu method [33] with modifications. For this purpose, a 0.1 mL methanol extract was diluted with 6.0 mL of distilled water, and then 0.5 mL of Folin–Ciocalteu reagent was added. After 3 min, 1.5 mL of sodium carbonate (7.5% *w*/*v*) was added and topped up with distilled water to 10 mL. The reaction mixture was stored for 30 min in a water bath (Memmert, Germany) at 40 °C. Absorbance was measured at λ = 765 nm. Results were expressed as mg gallic acid equivalent (GAE)/g extract. 

### 2.11. DPPH Free Radical Scavenging Activity

The ability of the analyzed samples to scavenge DPPH free radicals was evaluated according to Gangopadhyay et al. [32] with slight modifications: 2 mL of DPPH methanolic solution (0.06 mM) was added to 1 mL of a methanolic extract of the sample, mixed for 30 s and then incubated in the dark at room temperature for 20 min. The absorbance at 517 nm (UV-1800 Shimadzu, Kyoto, Japan) against methanol was measured. For blank, all reagents were added except the sample solution which was replaced by methanol (80%). The DPPH radical-scavenging activity (% inhibition) was calculated using Equation (4):

(4) $$\mathrm{Inhibition}\;\%=\frac{\left(A_{\mathrm{blank}}-A_{\mathrm{sample}}\right)}{A_{\mathrm{blank}}}\cdot 100$$

where *A*_blank_ was the absorbance of blank (without extract) and *A*_sample_ was absorbance of tested samples.

### 2.12. Consumer Acceptance 

The consumer acceptance of muffins with marigold and perilla extracts was carried out using a hedonic scale from 1 to 10, where 1 meant “I disliked extremely” and 10 meant “I liked extremely” according to Karp et al. [4]. The softness—S, moisture—M, porosity—P, taste—T, smell—SL, extract’s perceptibility—EP, crumb color—CbC, crust color—CtC, and overall quality—OQ were assessed by a group of 30 trained panelists from the Warsaw University of Life Sciences. All subjects were tested under identical conditions on the same day. Thirty identical sets of seven samples were prepared for evaluation. Approximately 15 g of each type of muffin was weighed and then placed in equal order on each test panel. All of the panelists were familiar with valuable by-products from bakery products. The samples were presented to them on the signed plates, and the panelists were given still water to use between the samples.

### 2.13. Statistical Analysis

Statistical analysis was performed using the Statistica 13.3 program (StatSoft Inc., Tulsa, OK, USA). The results of the experiments were used as variables and analyzed by one-way analysis of variance (ANOVA) using Fisher’s LSD test with the least significant difference at the significance level of α = 0.05.

## 3. Results and Discussion 

### 3.1. Characteristics of Selected Properties of MEx and PEx Extracts

According to the information obtained from the manufacturer, the MEx and PEx extracts were spray dried. Table 2 shows the color coordinates in the L*a*b* system and OHC of the marigold (MEx) and perilla (PEx) extracts. Color, as one of the significant determinants of food quality, is identified, inter alia, with freshness and allows consumers to choose food products, also thanks to the addition of substances that have a positive effect on human health. However, the addition of bioactive substances does not always positively affect the color of the final product. Analyzing the values of individual color components in the L*a*b* system of the plant extracts used, a higher lightness L* (*p* ≤ 0.05) was characteristic for the PEx in relation to the MEx. The MEx was characterized by redness (a*) and more than two times lower yellowness (b*) compared to PEx (6.19 vs. 22.41; *p* ≤ 0.05 and 18.82 vs. 44.28; *p* ≤ 0.05, respectively). According to Siriamornpun et al. [13], the color coordinates of the *Tagetes erecta* L. flower differed depending on the drying method used. The color components of fresh marigold flowers (L * = 83.2, a * = −3.4, b * = 66.8) in relation to this study had higher L * and b * values, which was probably due to the drying method used. Both the time and the method of drying used have a significant effect on the color components [34]. PEx tested in this study was characterized by high values of color components, which may result from the different distribution of pigments in plant parts, and also due to the use of spray drying. The advantage of spray drying is the very short exposure time of the product to high temperature [35]. From the point of view of using plant extracts for muffins, the oil-binding capacity is important, which facilitates the addition of the extract to the batter with a significant proportion of fat and can act as an emulsifier. In addition, the OHC is associated with flavor retention [36]. Significant differences in the oil binding capacity were noted between plant extracts (*p* ≤ 0.05). MEx increased its mass more than four times and was characterized by an almost 2 times higher OHC index in relation to PEx (*p* ≤ 0.05). OHC is often associated with the ability to maintain product moisture or in the case of meat products juiciness. In contrast, Aziah and Komathi [36] showed OHC for wheat flour 0.70 (g/g), for peeled pumpkin pulp flour 1.07 (g/g), and for unpeeled pumpkin pulp flour 1.36 (g/g). However, in the case of the Moringa oleifera leaf protein extract, the OHC was 3.55 (g/g) [36]. Food materials with high OHC can perform the function of functional ingredients. A product with high OHC can be a stabilizer of high-fat food matrices and is a good emulsifier. In bakery products, high OHC strengthens the protein–starch complex and supports the process of aerating the dough [37]. In a study by Varastegani et al. [38], the OHC of papaya pulp flour was compared with the OHC of wheat flour (2.47 vs. 1.05 (g/g), respectively). They observed a higher OHC of papaya flour relative to wheat flour, which was similar to the OHC of PEx used in this study. The authors indicate that the differences in OHC were due to the chemical and physical properties of the products analyzed [38].

### 3.2. Cooking Yield 

The cooking yield of baking muffins depending on the type and amount of vegetable extract used is shown in Table 3. It is related to the evaporation of water from the product and depends on the amount of substances dissolved in it [39]. The cooking yield of all samples with added extracts, as well as for the control sample, was over 80%. The addition of MEx and PEx significantly affected cooking yield (*p* ≤ 0.05), indicating that the extracts reduced water migration from the product. The MEx_1 sample had the highest cooking yield (86.7%), but at a comparable level with the other MEx and PEx samples (*p* > 0.05). The use of MEx and PEx at levels as low as 1% significantly reduced water migration, but further increases in the amount of extracts used did not result in a further reduction in water migration. In a study by Karp et al. [5], muffins with different cocoa fiber contents and sweetener levels (sucrose and steviol glycosides) showed higher cooking yields compared to the results of this study, which could be due to the different recipes and process parameters used.

### 3.3. Specific Volume

The volume of confectionery products is one of the factors determining desirability among consumers. Often, low-volume products are associated with compact, undercooked dough. PEx_5 samples had the highest specific volume (1.54 cm^3^/g), while MEx_3 and MEx_5 samples had the lowest (1.26 cm^3^/g) (Table 3). The type and amount of plant extract used did not cause changes in specific volume (*p* > 0.05). No significant change in the muffins’ specific volume may be due to the effect of not enough extract addition on the consistency and viscosity of the dough (similar retained air bubbles and CO_2_). In addition, the presence of egg foam in the recipe may compensate for the weakening of the gluten–starch mesh. In studies by Silva et al. [40], 1%, 3%, and 5% of ground perilla seeds were added to wheat bread. A decrease in specific volume was observed from 5.13 cm^3^/g (the control sample without the perilla addition) to 3.86 cm^3^/g. The reduced value of the product’s specific volume was due to the weakening of the gluten–starch matrix by limiting its development as a result of the addition of perilla seed meal used. These results were different from this work due to the recipe composition (yeast and bread flour were used) and different thermal processing conditions (temperature and time). Additionally, the use of different parts of the plant to produce the extract may have differed in the proportion of individual components (proteins, fats, and carbohydrates) in relation to the extract used in this study. In a study by Mamat et al. [3], the substitution of wheat flour with seaweed powder also resulted in a significant reduction in the muffins’ specific volume compared to the control sample. The difference in the effect on specific volume may have been due to the presence of carrageenan in the seaweed powdered material.

### 3.4. Water Activity 

Water activity (A_w_) is an important indicator of food shelf life and strongly influences the growth of microorganisms in food. It expresses the content of unbound / available or free water in food products. Increased Aw reduces the microbial stability of the product, determines the catalytic capacity and activity of enzymes, and affects the course of chemical reactions such as lipid oxidation and non-enzymatic browning [41,42]. Among all the samples, the lowest water activity of MEx_1 was 0.914 (*p* ≤ 0.05) (Table 3). The crumb of sponge cake (muffins) for all PEx samples was characterized by similar water activity (0.931 < A_w_ < 0.949; *p* > 0.05) to the control sample (A_w_ = 0.948). The water activity of food products ranges from 0.0 to 1.0, whereas foods from 0.9 to 1.0 are described as moist. At a water activity level of 0.89 to 0.55, foods are characterized by medium water content, and below 0.55 foods are classified as those with low water content. The A_w_ levels allow the grouping of foods into a specific category and predicting of the extent to which quality changes will occur, especially microbial growth dependent on the optimal A_w_ level (for most microorganisms in the range of 0.990–0.995) [41]. Therefore, with this type of product, the appropriate type of packaging, such as modified atmosphere packaging (as in the publication by Wyrwisz et al. [31]), must be considered to ensure quality and microbiological stability during storage and distribution. The results of muffins in the study by Heo et al. [43] also showed similar results to this study, where the A_w_ of the control samples and with a 4% share of Chinese cabbage leaf fiber were 0.94 and 0.93, respectively. In the case of using black carrot fiber concentrate in eggless rice muffins with xanthan gum, water activity decreased as the proportion of black carrot fiber increased to 0.86. This could indicate high water absorption and consequently limited free water availability [42]. In this study, no such trend was observed, which proves that MEx and PEx did not contain substances responsible for binding and holding water in the product.

### 3.5. Texture

Food texture influences the behavior of food in the mouth, and thus the perception and satisfaction with food quality [44]. To evaluate the textural properties of the developed muffins depending on the addition of plant extracts, a texture profile analysis (TPA) was conducted. The effects of the addition of MEx and PEx on changes in the texture of the muffins are presented in Table 3. The highest firmness [N] was characterized by MEx_1 (6.69), but at the same level as the control sample and PEx_1 (*p* ≤ 0.05), indicating that the 1% addition of extracts did not cause changes in firmness. The MEx_3 sample had the lowest firmness value (4.06) and was not statistically different between MEx_5, PEx_3, and PEx_5. Thus, it was observed that a greater addition of extracts did not reduce hardness and its stabilization. In the case of the study by Yoon et al. [45], who fortified muffins with freeze-dried *Perilla frutescens* leaf extract, an increase in muffin firmness was noted as a result of replacing flour; however, differences for this texture parameter were not significantly different with the control sample. The results of this study on the addition of PEx were similar to the study of Yoon et al. [45]. The muffins enriched with paprika pomace powder [46] and ground Matcha green tea [9] showed the opposite trend of firmness change. The reason of decreasing firmness may have been the increasing water content of the product, as noted by conducting a water absorption analysis for Matcha tea powder extracts [9]. The direction of changes in the springiness of the muffins depended on the type of plant extract used. Both 1% addition of MEx and PEx increased the springiness of the product. Compared to the control sample, muffins with MEx addition at 3 and 5% were characterized by a decrease in muffin springiness (*p* ≤ 0.05). The lowest springiness value was characterized by MEx_5 (0.4). For samples with PEx, an increase in springiness was observed with an increase in the proportion of extract in the sample compared to the control sample (*p* ≤ 0.05). The studies conducted by Yoon et al. [45] showed different results, which showed no significant differences in the effect of the concentration of freeze-dried perilla leaves extract on muffin springiness. In the case of texture studies conducted in gluten-free muffins with dried berries, higher springiness was noted [47] in relation to this study for the control sample and with MEx. In contrast, only muffins with PEx had a similar springiness to the samples obtained in the study by Kowalczewski and Ivanišová [47]. The cohesiveness of the samples increased with the level of extract addition in the muffins; however, from MEx_3 and for PEx_5 the changes were statistically significant (*p* ≤ 0.05). The control sample (0.38), PEx_1 (0.39), PEx_3 (0.40) samples had the lowest value for this texture parameter. In contrast, the significantly highest cohesiveness was obtained from the MEx_3, MEx_5, and PEx_5 samples (0.43) (*p* ≤ 0.05).

### 3.6. Color 

The lightness of the crust is one of the factors that allows the consumer to determine the quality of the product at the purchase stage. A crust that is too dark and overcooked may indicate that the heat treatment conditions used were not optimal. This parameter, too, on the other hand, can indicate the perception of a product’s nutritional value, since dark products are associated with the use of whole grains in the production of cereal products. Among all samples, PEx_1 and PEx_3 muffins had the lowest crust coordinate value L* (23.84 and 24.28, respectively) and were significantly darker than the control sample (Table 4). The lightness L* of the crust for muffin samples with MEx regardless of the amount added was similar to the control sample. The shortcake cookies made with perilla leaf powder in the study by Choi et al. [48] also differed significantly in their level of color lightness compared to the control sample. The muffins analyzed in this work were characterized by lower crust lightness. The differences in lightness for the cookies in the study cited above [48] may have been due to the shorter time (12 min) and lower baking temperature (170 °C). In the study by Nath et al. [46], the lightness of muffin crusts with the addition of dry paprika pomace powder was twice as high as in this study [46]. Muffin crusts enriched with kale powder were also characterized by a similar degree of brightness [49]. According to Gomez et al. [50], the color of the muffin crust formed during the baking process essentially depends on the course of the Millard reaction between the sugars and amino acids in the product and as a result of the caramelization of the sugars. Partial replacement of flour in the recipe with plant extracts can cause changes in the proportion of proteins and carbohydrates, which can result in a different course of the Millard reaction. The redness (a*) of the muffin crust with different amounts of plant extracts is presented (Table 4). The addition of MEx did not significantly affect the redness, regardless of its amount (*p* > 0.05) compared to the control sample. In contrast, the addition of PEx reduced the redness a* in the muffin compared to the control sample (*p* ≤ 0.05), with no significant effect of the amount of this extract on changes in this coordinate (*p* > 0.05). In a study by Choi et al. [48], the value of the a* component of the crust of shortcake determined changes in greenness, which increased with increasing amounts of freeze-dried perilla powder. This is related to the color of the raw material used for the extract. The direction of red color changes for MEx samples was similar to the results obtained in the study by Nath et al. [46] due to the presence of carotenoids in the powdered extracts, which determine such color. The addition of plant extracts caused changes in the yellowness (b*) of the samples (Table 4). The surface of MEx_1 and MEx_3 muffins showed similar yellowness to the control sample (*p* > 0.05). In contrast, the MEx_5 sample had the highest yellowness b* (24.20) (*p* ≤ 0.05). There was a significant effect of the PEx additive regardless of its amount on reducing the yellowness compared to the control sample (*p* ≤ 0.05). The study by Nath et al. [46] showed a similar trend of yellowness changes to samples using MEx due to the presence of carotenoids in red pepper extract. In the case of muffins in the study by Yoon et al. [45], the use of powder from freeze-dried *Perilla frutescens* leaves resulted in a reduction in yellowness b*. The direction of change was consistent with the results obtained in this study. The values of the overall color difference ΔE with the extract and control samples were smaller with MEx than with PEx. The ΔE for the MEx samples ranged from 2.88–5.13, while that of the PEx samples ranged from 9.81–13.05. Nevertheless, these were already noticeable differences in color by the observer. There was a significant effect of the addition of extracts, regardless of the type of extract and its quantity, on the color components in the L* a*b* system of the muffin crumb (*p* ≤ 0.05) (Table 4). The lightest crumb was the control sample (61.63) (*p* ≤ 0.05), and the sample with PEx_5 (29.26), indicating a decrease in lightness by almost half (*p* ≤ 0.05). MEx did not cause a change in crumb lightness, while PEx caused a significant change starting at 3% of this extract compared to the control sample. As shown in the study of Sabanis et al. [51], the temperature of the baking process did not affect the color of the crumb. The authors reasoned that the color components, including the lightness of the crumb, may have depended mainly on the color of the additive used as a flour substitute since the crumb did not reach as high a temperature as the crust during the baking. There was also a significant effect of the addition of plant extract on the redness (a*) of the crumb of sponge cake (muffins) (*p* ≤ 0.05). The crumb of MEx_5 muffins had the highest redness (15.09) compared to the control sample (3.37) (*p* ≤ 0.05). In addition, it was shown that the increase in the value of coordinate a* for MEx samples was directly proportional to the amount of extract in the product. The crumb of muffins with a partial proportion of red pepper pomace powder) showed a similar direction of change in red color saturation (a*), indicating the significant influence of plant products rich in pigments such as carotenoids on crumb color [46]. For samples with PEx, it was shown that increasing the amount of this extract above 3% no longer increased in the redness of the product (*p* > 0.05). Analyzing the values of the b* crumb parameter, it was noted that for the samples with MEx, the yellow crumb color values were significantly higher with respect to the control sample (*p* ≤ 0.05). For muffins from PEx, there was a significant reduction in yellowness compared to the control sample (*p* ≤ 0.05). The PEx_3 and PEx_5 samples had the lowest value of the b* component (*p* ≤ 0.05). The overall color difference ΔE between the samples with extracts and the control sample was very high (18.03 < ΔE < 34.85) and increased with the amount of extract added, regardless of the type of extract. Such high ΔE values indicate that the differences could be identified by an untrained observer.

### 3.7. Total Polyphenol Content

**Figure 1:**
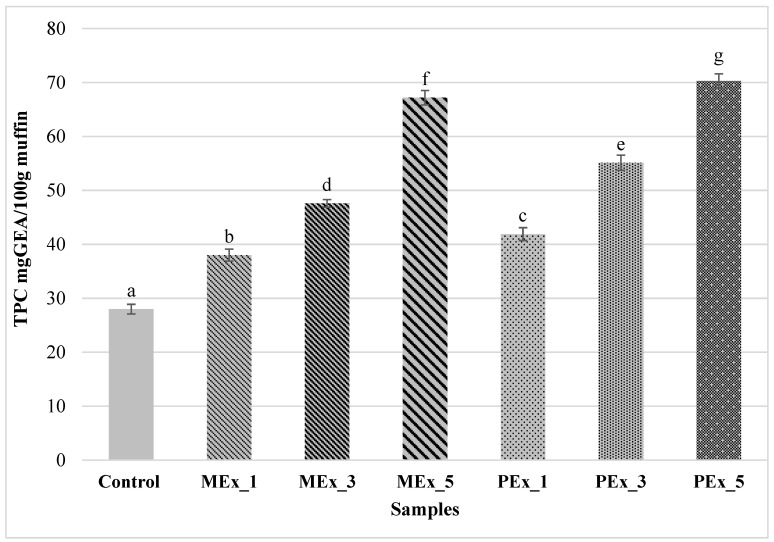
Mean ± SD for total polyphenol content (TPC) in muffins. MEx_1, MEx_3, and MEx_5—samples with 1, 3, and 5% replacement of flour on dry marigold extract, respectively. PEx_1, PEx_3, and PEx_5—samples with 1, 3, and 5% replacement of flour on dry perilla extract, respectively. ^a, b, c, d, e, f, g^—means with different letters are significantly different (test LSD: *p* ≤ 0.05).

The total polyphenol content (TPC) of samples with extracts increased proportionally to the amount of extracts added (Figure 1). Of the samples tested, the control sample had the lowest TPC content (27.99; *p* ≤ 0.05). After using both MEx and PEx, the highest TPC values were represented by muffin samples with the highest addition of extracts in the formulation: MEx_5 and PEx_5 (67.19 and 70.29, respectively). As the amount of extract in the product increased, TPC increased, but the values were significantly higher (*p* ≤ 0.05) for samples with PEx addition. Similar changes in TPC content were observed in muffins fortified with pomegranate fruit peel powder [52], where an increase in polyphenol content was observed as the proportion of pomegranate peels increased. According to the authors of the study, the increasing TPC content was due to the higher antioxidant value of pomegranate peels in relation to the TPC content of the flour used. In a study by Siriamornpun et al. [13], TPCs for marigold flower extracts prepared by various drying methods including freeze-drying were characterized by TPCs in the range of 43.6–60.0 mg GAE/g DM. These results were significantly higher for the TPCs contained in the muffins in this work, which may indicate partial degradation of antioxidant-like compounds during the muffin baking process. In muffins prepared using dry perilla powder in amounts of 0.5%, 1%, 3%, and 5%, a concentration-dependent increase in TPC content in the recipe was noted [48]. Muffins with 5% addition of freeze-dried perilla leaves contained nearly 140 mg GAE/100 g, while PEx_5 muffins had TPC = 70.29 mg GAE/100 g. This may be due to the fact that the baking temperature of the muffins was lower (170 °C) and the baking time was shorter (12 min). In addition, the differences in TPC may be related to the use of extracts from different morphological parts of plants, and different species, whose growing conditions, processing, storage, and application methods may have been different.

### 3.8. DPPH Free Radical Scavenging Capacity

**Figure 2:**
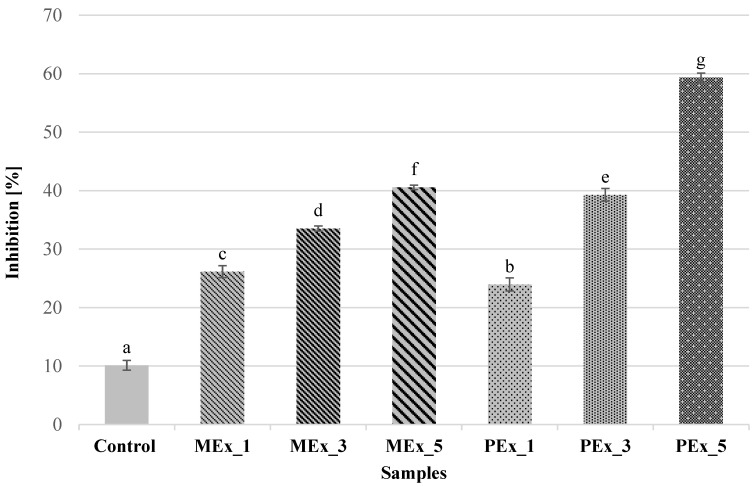
Mean ± SD for DPPH free radical scavenging capacity. MEx_1, MEx_3, and MEx_5—samples with 1, 3, and 5% replacement of flour on dry marigold extract, respectively. PEx_1, PEx_3, and PEx_5—samples with 1, 3, and 5% replacement of flour on dry perilla extract, respectively. ^a, b, c, d, e, f, g^—means with different letters are significantly different (test LSD: *p* ≤ 0.05).

The values of DPPH free radical scavenging capacity are shown in Figure 2, which ranged from 10% to nearly 60%. There was a significant effect of the addition of MEx and PEx on the free radical scavenging capacity (*p* ≤ 0.05), depending on the size of the extract share in the sample. As the proportion of extract in the sample increased, the free radical scavenging capacity of DPPH* increased. It can be seen that DPPH was positively correlated with TPC content in the tested muffins. The control sample had the lowest antioxidant capacity (10.14%), and PEx_5 (59.33%) had the highest (*p* ≤ 0.05). Considering the two different extracts used, with a 1% addition of extracts, the DPPH* free radical scavenging capacity was higher in MEx_1 relative to PEx_1, while the subsequent samples (MEx_3 and MEx_5) showed lower DPPH* free radical scavenging capacity relative to PEx_3 and PEx_5 (*p* ≤ 0.05). Muffins using freeze-dried leaves of the perilla plant (*Perilla frutescens var. japonica HARA*) in a study by Yoon et al. [45] showed similar antioxidant potential. As the proportion of *Perilla frutescens* powder increased, the hydrogen-donating capacity increased. It can be noted higher values for the control sample in relation to the results of this work (14.65% vs. 10.14%), while the proportion of 3% addition of freeze-dried extract from the study of Yoon et al. [44] in relation to the results obtained was lower (33.84%, 39.23%, respectively). This difference may be due to the use of different methods for obtaining extracts (freeze-drying vs. spray-drying). A similar trend of changes in DPPH free radical scavenging capacity was observed in a study by Choi [49]. Muffins enriched with kale powder at 3%, 6%, 9%, and 12% had a DPPH radical scavenging activity * of 25.70% in the control group, while it ranged from 34.80 to 53.70% in samples with kale powder. This shows that MEx has comparable and PEx higher DPPH free radical scavenging capacity than kale extract.

### 3.9. Consumer Acceptance 

**Figure 3:**
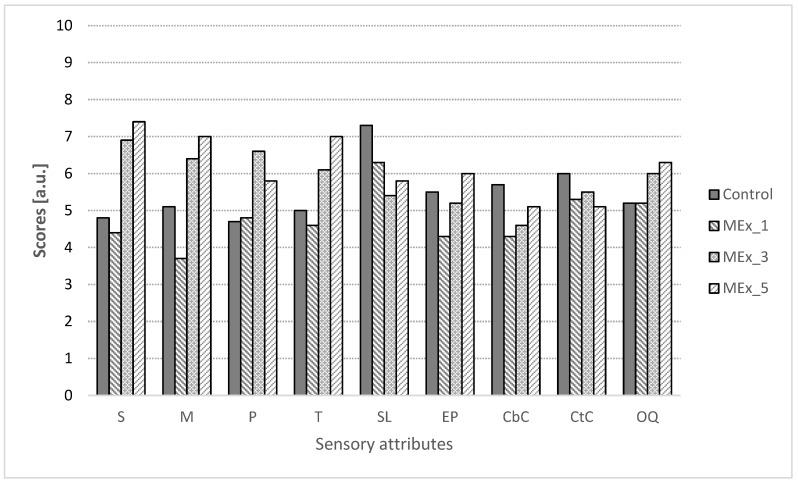
Scores of consumer acceptance analysis of muffins with dry Mex. MEx_1, MEx_3, and MEx_5—samples with 1, 3, and 5% replacement of flour on dry marigold extract, respectively. S—softness, M—moisture, P—porosity, T—taste, SL—smell, EP—extract’s perceptibility, CbC—crumb color, CtC—crust color, OQ—overall quality.

**Figure 4:**
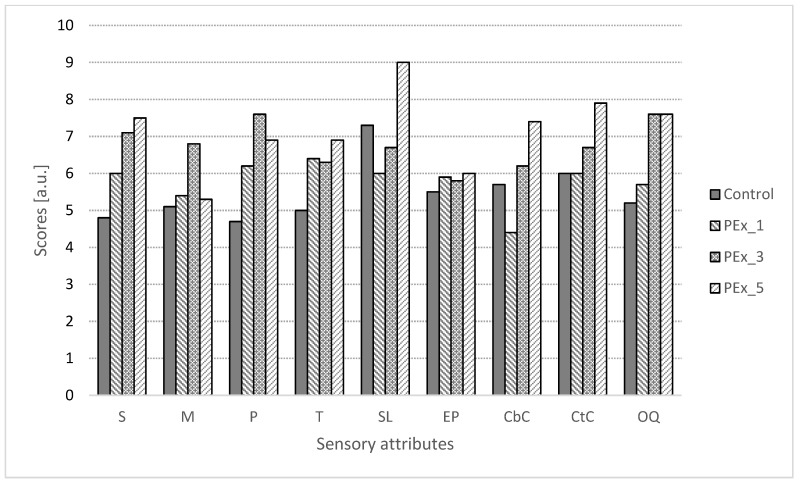
Scores of consumer acceptance analysis of muffins with dry PEx. Pex_1, Pex_3, and Pex_5—samples with 1, 3, and 5% replacement of flour on dry perilla extract, respectively. S—softness, M—moisture, P—porosity, T—taste, SL—smell, EP—extract’s perceptibility, CbC—crumb color, CtC—crust color, OQ—overall quality.

In Figure 3 and Figure 4, the consumer acceptance scores selected sensory attributes of muffins with MEx and PEx are shown. Taking all samples into consideration, it can be said that the addition of MEx and PEx improved the sensation of evaluated attributes. Softness and moisture content of all samples were rated higher than the control sample except for MEx_1. The softness of the muffins with the highest proportion of extracts (MEx_5 and PEx_5) was the highest rated among consumers. When considering porosity, the highest ratings were given to the MEx_3 and PEx_3 samples (6.6 and 7.6, respectively). In addition, muffin flavor was rated higher in samples with extracts relative to the control sample except for MEx_1. The smell of the samples was evaluated differently, where only PEx_5 obtained very high notes relative to the control sample, while the other samples had lower scores. The extract’s desirability was rated similarly; however, the presence of plant extracts (except MEx_1 and MEx_3) resulted in higher desirability compared to the control sample. The color of the muffin crust was rated higher than that of the crumb. The crust color of the PEx_5 sample scored close to 8 points, which may have been due to their similarity to popular chocolate muffins. Of the seven samples, muffins from PEx_5 obtained the highest overall quality rating and received an average score of 7.6. Muffins from MEx_3 and MEx_5 received equally high ratings. Of all the samples, the lowest notes for overall quality were given to the control sample.

It is noteworthy that in the present study, the plant extract addition resulted not only in an increase in total polyphenol content and total antioxidant capacity in the product, but it also did not negatively affect the selected physical characteristics of the muffins. Furthermore, the addition of MEx and PEx had a positive effect on the muffins’ sensory characteristics compared to the control sample. 

## 4. Conclusions

The study showed that the partial replacement of flour in a sponge cake recipe with perilla or marigold extract can be a good alternative to classic products of this type. In addition to the contributed potential, which may be health-promoting, muffins have proven to be more desirable in terms of attributes such as taste, smell, porosity, and color in relation to the classic product. The antioxidant capacity of the muffins increased proportionally with the concentration of the extract in the product. Muffins with marigold extract in the recipe had lower antioxidant capacity than those with perilla extract. Muffins, which are a popular product readily consumed by consumers, can be a good carrier of health-promoting substances while providing a tasty product with a rich flavor and aroma profile thanks to the use of plant extracts. In particular, muffins with perilla extract can be a good alternative to the classic muffin, as confirmed by the results of the consumer analysis. 

## Figures and Tables

**Table 1:** Composition of used muffin formulations expressed on a 100 g of flour or 100 g of flour with extract basis.

Ingredients [g]	Control	MEx_1	MEx_3	MEx_5	PEx_1	PEx_3	PEx_5
Wheat flour (type 450)	100.0	96.6	89.8	83.0	96.6	89.8	83.0
Sugar	38.6	38.6	38.6	38.6	38.6	38.6	38.6
Rapeseed oil	30.0	30.0	30.0	30.0	30.0	30.0	30.0
Yogurt	105.4	105.4	105.4	105.4	105.4	105.4	105.4
Baking powder	4.8	4.8	4.8	4.8	4.8	4.8	4.8
Eggs	52.0	52.0	52.0	52.0	52.0	52.0	52.0
Marigold extract (MEx)	-	3.4	10.2	17.0	-	-	-
Perilla extract (PEx)	-	-	-	-	3.4	10.2	17.0
Salt	0.5	0.5	0.5	0.5	0.5	0.5	0.5

MEx_1, MEx_3, and MEx_5—samples with 1, 3, and 5% replacement of flour on dry marigold extract, respectively. PEx_1, PEx_3, and PEx_5—samples with 1, 3, and 5% replacement of flour on dry perilla extract, respectively.

**Table 2:** Mean ± SD for color coordinates in CIE L*a*b* and oil holding capacity (OHC) of MEx and PEx.

Extracts	Color in CIE L*a*b*			OHC [g/g]
	L*	a*	b*	
MEx	54.65 ± 0.01 ^a^	6.19 ± 0.03 ^a^	18.82 ± 0.02 ^a^	4.4 ± 0.11 ^b^
PEx	71.12 ± 0.61 ^b^	22.41 ± 0.29 ^b^	44.28 ± 0.93 ^b^	2.5 ± 0.08 ^a^

Mex—dry marigold extract; Pex—dry perilla extract. ^a, b^—means with different letters within the same column are significantly different (test LSD: *p* ≤ 0.05).

**Table 3:** Mean ± SD of cooking yield, specific volume, water activity, and selected texture components of muffins.

Samples	Cooking Yield [%]	Specific Volume [cm^3^/g]	Aw	Firmness [N]	Springiness [-]	Cohesiveness [-]
Control	81.71 ± 1.14 ^a^	1.41 ± 0.21 ^a^	0.948 ± 0.005 ^b^	6.25 ± 0.39 ^b^	0.55 ± 0.07 ^b^	0.38 ± 0.04 ^a^
MEx_1	86.89 ± 0.65 ^b^	1.46 ± 0.27 ^a^	0.914 ±0.014 ^a^	6.69 ± 0.48 ^b^	0.60 ± 0.05 ^b^	0.41 ± 0.01 ^ab^
MEx_3	85.52 ± 0.74 ^b^	1.26 ± 0.17 ^a^	0.953 ± 0.006 ^b^	4.06 ± 0.61 ^a^	0.45 ± 0.07 ^a^	0.43 ± 0.02 ^b^
MEx_5	85.71 ± 0.37 ^b^	1.26 ± 0.20 ^a^	0.936 ± 0.002 ^ab^	4.29 ± 0.82 ^a^	0.35 ± 0.04 ^a^	0.43 ± 0.03 ^b^
PEx_1	85.16 ± 0.89 ^b^	1.45 ± 0.27 ^a^	0.947± 0.016 ^b^	6.60 ± 0.46 ^b^	0.66 ± 0.06 ^c^	0.39 ± 0.03 ^a^
PEx_3	85.58 ± 0.93 ^b^	1.24 ± 0.17 ^a^	0.931 ± 0.028 ^ab^	4.36 ± 0.54 ^a^	0.70 ± 0.05 ^c^	0.40 ± 0.03 ^a^
PEx_5	85.54 ± 0.64 ^b^	1.54 ± 0.21 ^a^	0.939 ± 0.006 ^b^	4.18 ± 0.62 ^a^	0.73 ± 0.03 ^c^	0.43 ± 0.00 ^b^

MEx_1, MEx_3, and MEx_5—samples with 1, 3, and 5% replacement of flour on dry marigold extract, respectively. PEx_1, PEx_3, and PEx_5—samples with 1, 3, and 5% replacement of flour on dry perilla extract, respectively. ^a, b, c^—means with different letters within the same column are significantly different (test LSD: *p* ≤ 0.05).

**Table 4:** Mean ± SD of crust and crumb color coordinates (L*a*b*) and ΔE of muffins.

	Crust				Crumb			
	L*	a*	b*	ΔE	L*	a*	b*	ΔE
Control	29.10 ± 1.92 ^c^	11.96 ± 1.16 ^c^	19.42 ± 1.51 ^b^	-	61.63 ± 2.17 ^d^	3.37 ± 0.39 ^a^	25.63 ± 0.87 ^c^	-
MEx_1	29.97 ± 2.05 ^c^	10.22 ± 1.59 ^bc^	17.29 ± 2.68 ^b^	2.88	43.92 ± 2.40 ^c^	7.09 ± 0.36 ^b^	44.01 ± 2.05 ^e^	25.79
MEx_3	29.24 ±1.73 ^c^	11.44 ± 1.79 ^c^	16.40 ± 1.93 ^b^	3.07	41.23 ± 1.95 ^c^	9.82 ± 0.77 ^e^	42.34 ± 1.95 ^de^	27.14
MEx_5	28.27 ± 2.51 ^bc^	13.63 ± 1.84 ^c^	24.20 ± 2.64 ^c^	5.13	42.72 ± 2.91 ^c^	15.09 ± 1.02 ^f^	42.72 ± 3.63 ^de^	28.05
PEx_1	23.84 ± 1.50 ^a^	7.46 ± 1.26 ^a^	12.47 ± 1.74 ^a^	9.81	44.46 ± 2.28 ^c^	5.05 ± 0.12 ^b^	20.39 ± 0.88 ^b^	18.03
PEx_3	24.28 ± 1.86 ^a^	6.79 ± 2.12 ^a^	8.45 ± 2.73 ^a^	13.05	33.78 ± 2.06 ^b^	6.13 ± 0.66 ^c^	15.86 ± 2.27 ^a^	29.64
PEx_5	27.14 ± 1.98 ^b^	8.24 ± 1.63 ^ab^	8.91 ± 0.99 ^a^	11.32	29.26 ± 1.92 ^a^	6.13 ± 0.63 ^c^	13.00 ± 1.60 ^a^	34.85

MEx_1, MEx_3, and MEx_5—samples with 1, 3, and 5% replacement of flour on dry marigold extract, respectively. PEx_1, PEx_3, and PEx_5—samples with 1, 3, and 5% replacement of flour on dry perilla extract, respectively. ^a, b, c, d, e, f^—means with different letters within the same column are significantly different (test LSD: *p* ≤ 0.05).

## Data Availability

Not applicable.
